# Fungal effectors versus defense-related genes of *B. juncea* and the status of resistant transgenics against fungal pathogens

**DOI:** 10.3389/fpls.2023.1139009

**Published:** 2023-06-08

**Authors:** Prajjwal Rai, Laxman Prasad, Pramod Kumar Rai

**Affiliations:** ^1^ Division of Plant Pathology, Indian Agriculture Research Institute, New Delhi, India; ^2^ Division of Plant Pathology, Directorate of Rapeseed-Mustard Research, Bharatpur, India

**Keywords:** *B. juncea*, fungal pathogens, effectoromics, resistance genes, quantitative trait loci, defense-related genes, transgenics

## Abstract

Oilseed brassica has become instrumental in securing global food and nutritional security. *B. juncea*, colloquially known as Indian mustard, is cultivated across tropics and subtropics including Indian subcontinent. The production of Indian mustard is severely hampered by fungal pathogens which necessitates human interventions. Chemicals are often resorted to as they are quick and effective, but due to their economic and ecological unsustainability, there is a need to explore their alternatives. The *B. juncea*-fungal pathosystem is quite diverse as it covers broad-host range necrotrophs (*Sclerotinia sclerotiorum*), narrow-host range necrotrophs (*Alternaria brassicae* and *A. brassicicola*) and biotrophic oomycetes (*Albugo candida* and *Hyaloperonospora brassica*). Plants ward off fungal pathogens through two-step resistance mechanism; PTI which involves recognition of elicitors and ETI where the resistance gene (*R* gene) interacts with the fungal effectors. The hormonal signalling is also found to play a vital role in defense as the JA/ET pathway is initiated at the time of necrotroph infection and SA pathway is induced when the biotrophs attack plants. The review discuss the prevalence of fungal pathogens of Indian mustard and the studies conducted on effectoromics. It covers both pathogenicity conferring genes and host-specific toxins (HSTs) that can be used for a variety of purposes such as identifying cognate *R* genes, understanding pathogenicity and virulence mechanisms, and establishing the phylogeny of fungal pathogens. It further encompasses the studies on identifying resistant sources and characterisation of *R* genes/quantitative trait loci and defense-related genes identified in Brassicaceae and unrelated species which, upon introgression or overexpression, confer resistance. Finally, the studies conducted on developing resistant transgenics in Brassicaceae have been covered in which *chitinase* and *glucanase* genes are mostly used. The knowledge gained from this review can further be used for imparting resistance against major fungal pathogens.

## Introduction

The Brassicaceae family showcases tremendous diversity with approximately 3709-member species and 338 genera (Warwick et al., 2006).This group offers great economic significance with a range of utilities such as edible and industrial oil, condiments, and vegetables (Nesi et al., 2008). To establish evolutionary relationship between the members of Brassicaceae, Nagaharu (1935) gave the ‘triangle of U’ model, in which he proposed the three amphidiploid species, *Brassica napus* (AACC), *B. juncea* (AABB), and *B. carinata* (BBCC), evolved through the interspecific hybridization in nature between diploid species *B. rapa* (AA), *B. nigra* (BB), and *B. oleracea* (CC). *B. rapa* and *B. napus* are important in temperate countries, while *B. juncea* dominates the subtropics such as the Indian subcontinent (OECD, 2016). *B*. *juncea* (L.) Czern & Coss (AABB, 2*n* = 4*x* = 36) traces its origin to *Brassica rapa* (AA, 2n = 20) and *Brassica nigra* (BB, 2n = 16). Axelsson et al. (2000) proved that both the parental genomes in *B. juncea* were conserved and have not undergone any change since polyploidization. A similar study was also carried out by Parkin and Lydiate (1997) in *B. napus* to establish the intact nature of parental genomes in it. Yang et al. (2016) sequenced 954.90 Mb genome of *B. juncea* and found that, the A subgenomes in *B. napus* and *B. juncea* have independent origins. Globally, Brassica or rapeseed-mustard is grown over 36.59 Mha with production and productivity of 72.37 Mt, and 1980 kg/ha, respectively. India’s global acreage and production share stood at 19.8% and 9.8% (USDA, 2020).

The *B. juncea* production is hampered by several biotic and abiotic stresses. Out of which, fungal diseases are a serious concern. The crop is vulnerable to fungal pathogens owing to the genetic uniformity between all prevalent cultivars. Alternaria leaf blight, White rust, Sclerotinia stem rot, and Downy mildew are the major fungal diseases of *B. juncea* (Singh et al., 2021; Meena et al., 2022). Though, chemicals are a good option for quick and effective control of diseases, they are unsustainable from both economic and ecological perspectives. For developing a robust disease management plan, the focus must shift from pathogen management to host management and there should be substantial efforts to alter crop ecology in the host’s favour (He et al., 2016). The specific host-pathogen interaction must be kept in mind for deciding the RAER (Resistance, Avoidance, Elimination, and Remediation) strategy (Xie et al., 1984). Genetic resistance is considered best to manage plant diseases as it is compatible with all other disease management strategies (Saharan, 1992; Saharan et al, 2005; Ren et al., 2016). The holy grail of resistance development in Brassica is identifying the pathogen virulence factors (effectors and toxins) and the complementary host *R* genes. In addition to these, the role of defense-related genes must be established as they are thought to contribute in broad-spectrum non-host resistance (NHR).

Plants have a two-step resistance mechanism against invading pathogens. The first one is the pattern triggered immunity (PTI), which is also known as basal resistance (Jones and Dangl, 2006). The pathogen-associated molecular patterns (PAMPs) (exogenous elicitors) are recognised by the pattern recognition receptors (PRRs), while wall-associated kinases (WAKs) detect the damage-associated molecular patterns (DAMPs) (endogenous elicitors) (Heil and Land, 2014). A plethora of defense responses is activated by PTI, which covers the influx of extracellular Ca^2+^ into the cytosol (Ranf et al., 2011), followed by the activation of MAP kinases (Zhang et al., 2007), reactive oxygen species (ROS) (Scheler et al., 2013), and other hormonal signaling molecules, such as salicylic acid, jasmonic acid, ethylene, and cytokinin (Jin et al., 2008, Nakano et al., 2013, Zhang et al., 2018; Huang et al., 2020).

**Figure 1:**
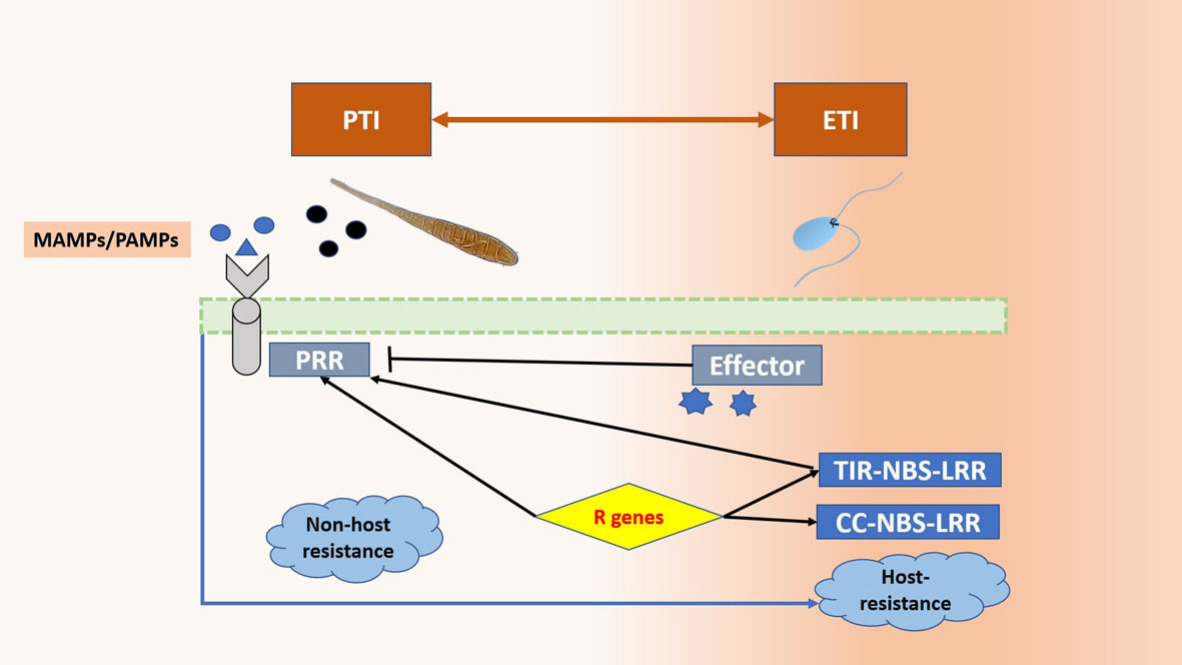
Two-step resistance mechanism in plants. The PAMPs/MAMPs are recognised by the PRRs. This leads to PTI (Pattern Triggered Immunity). To overcome this, the pathogen employs effectors and leads to ETS (Effector Triggered Susceptibility) and the plant responds by initiating ETI (Effector triggered immunity) in which the intracellular receptor recognises the effectors and leads to resistance.

**Figure 2:**
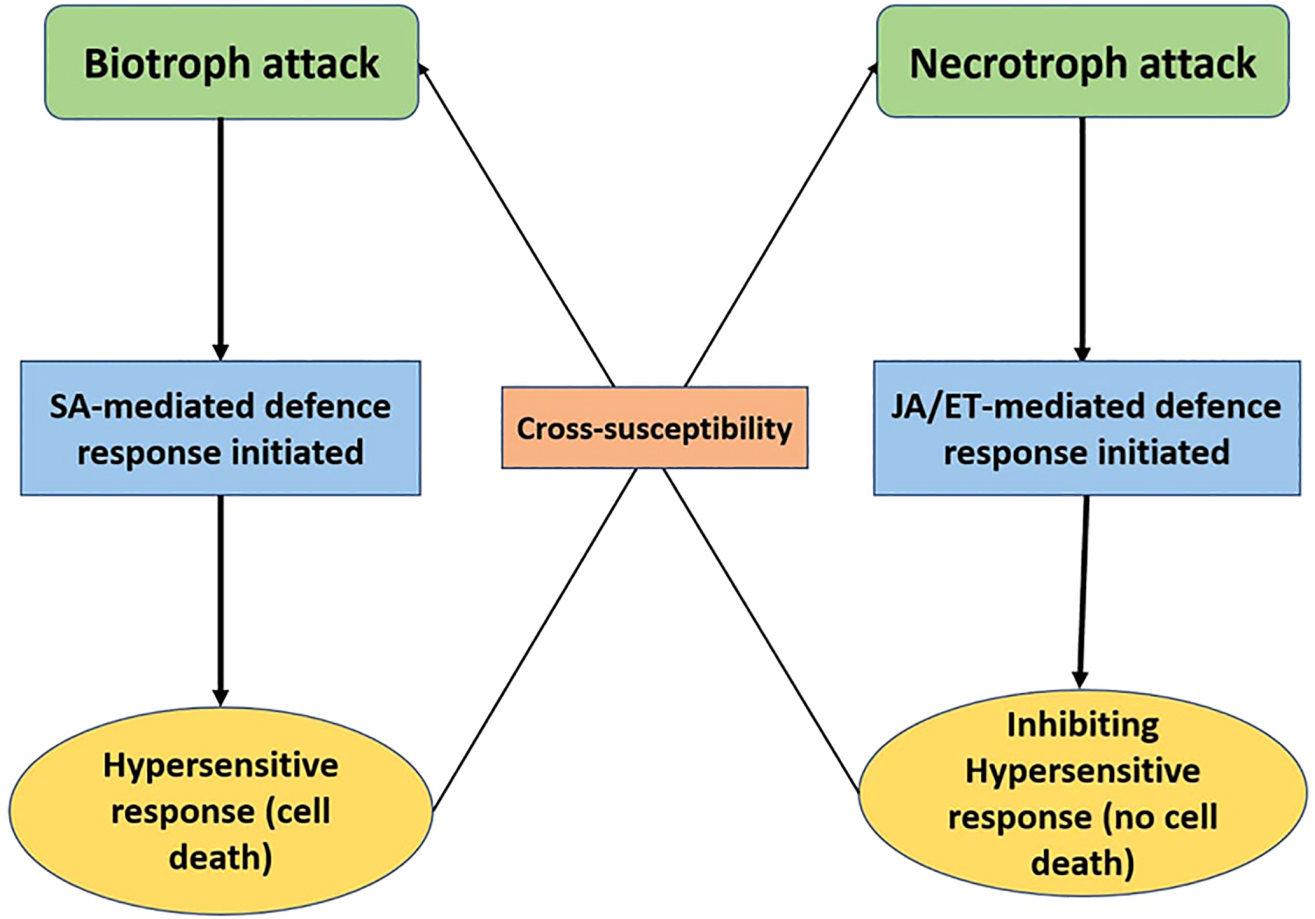
Schematic diagram of differential hormonal signalling defence response given at the time of attack by biotrophs and necrotrophs, where the SA pathway is initiated in the former and JA/ET in the latter.

This basal response is overcome by pathogen effectors, which results in effector triggered susceptibility. The plant fights back by recognising these effectors with intracellular receptors (*R* genes), thus activating the effector triggered immunity (ETI) (Peng et al., 2018; Alhoraibi et al., 2019). The host-pathogen interaction depends upon the trophic strategy of the pathogen. Biotrophs generally rely on the subtle manipulation of the host defenses for evading detection and effectors are the major weapons for this purpose. The plant’s response is characterised by the hypersensitive response (HR), which is a trusted ally against these pathogens as it limits the food source. The *Effector-R* gene interaction leads to ETI in the case of biotrophs. On the other hand, necrotrophs have a vast array of disease agents such as toxins, cell death inducing proteins (CDIPs), secondary metabolites, and CWDEs (cell wall degrading enzymes). Hypersensitive response against necrotrophs aids the pathogen instead and it is avoided by plants. The effector-intracellular receptor interaction is characterised by ETS for Necrotrophs (Laluk and Mengiste, 2010; Ghozlan et al., 2020; Liao et al., 2022). The difference in defense response has also been observed in terms of hormonal signaling pathways. The rise in salicylic acid (SA) levels increases resistance against biotrophs but it makes the plant vulnerable to necrotrophs (Caarls et al., 2015). The jasmonic acid (JA) pathway is antagonistic to SA and initiated at the time of attack by a necrotroph (Kravchuk et al., 2011; Niu et al., 2011; Weller et al., 2012) **Figure 1** (**Figure 2**).

A robust disease management strategy requires an understanding of the pathogenesis mechanisms which will enable identification of candidate effectors and disease resistance targets (Kim et al., 2016; Van de Wouw and Idnurm, 2019). The principle is based on the fact that resistant varieties will be insensitive to pathogen effectors. This functional assay can help to avoid tedious field trials and infection assays and further act as markers for fastening resistance breeding programs. Effector-based functional assays can be utilised for accelerating *R* gene cloning and finding out redundancy. It can help us to bring more specificity in breeding programs as the effector-based distinction of resistant sources is better than isolate-based (Du and Vleeshouwers, 2014). A Pathogen-Host Interactions database (PHI-base) can also be set up for analysis of phenotypic and biological data on virulence, pathogenicity, and effector proteins (Urban et al., 2017). Next step is the identification and utilisation of the resistant sources in cultivated and wild *Brassica*, which has been aided by the release of reference genome data of cultivated brassica species (Wang et al., 2011; Chalhoub et al., 2014; Liu et al., 2014; Parkin et al., 2014; Yang et al., 2016). Other economically viable and ecologically sustainable solutions such as healthy crop husbandry and biological control can be employed depending on their suitability in each pathosystem.

**Figure 3:**
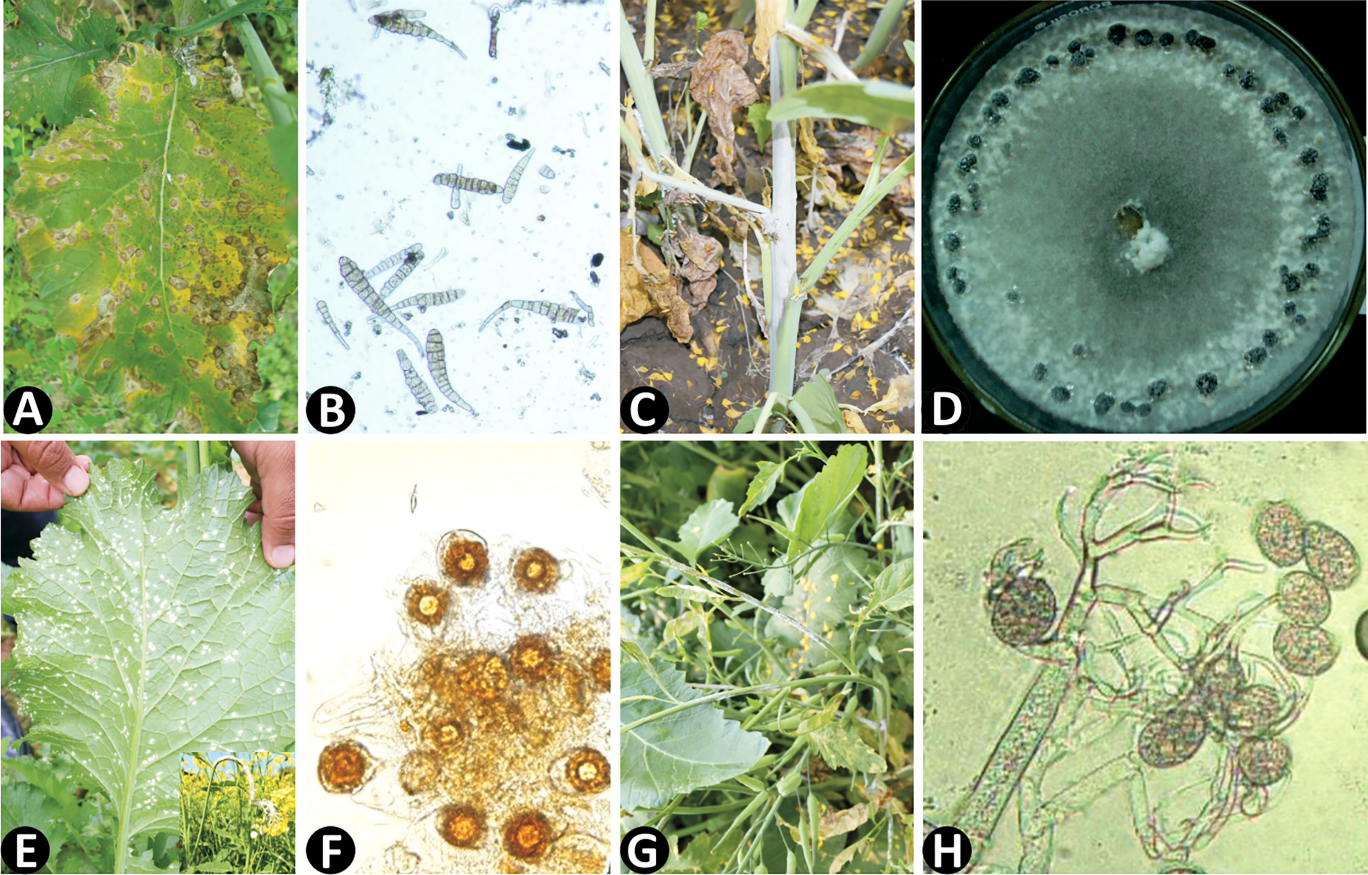
**(A)** Alternaria leaf blight symptons on Indian mustard (*B. juncea*); **(B)** Conidia of *Alternaria brassicae*; **(C)** Sclerotinia stem rot symptoms; **(D)** *Sclerotinia sclerotiorum* mycelium and sclerotia on PDA; **(E)** White rust on Indian mustard leaf (inset: hypertrophy); **(F)** Germinating oospores of *Albugo candida*; **(G)** Downy mildew on Indian mustard; **(H)** Sporangia and sporangiospores of *Hyaloperonospora brassicae*.

The present review aims to encompass the studies conducted on the 4 major fungal diseases of rapeseed-mustard with special emphasis on effectoromics of pathogens (**Table 1**), the sources of resistance genes/QTLs and defense-related genes identified across the Brassicaceae family (**Table 2**) and the transgenics developed for disease resistance (**Table 3**). Thus, this knowledge can be harnessed to improve Brassica against devastating fungal diseases such as Alternaria leaf blight (**Figures 3A, B**), Sclerotinia stem rot (**Figures 3C, D**), white rust (**Figures 3E, F**), and downy mildew (**Figures 3G, H**).

**Table 1:** The effectors identified and characterized in major fungal pathogens of the Brassicaceae family.

Pathogen	Effector	Reference
*Alternaria brassicae*	*Destruxin-B* *ABR-toxin* *AbrNLP1* and *AbrNLP2*	Bains and Tewari, 1987 and Buchwaldt and Green, 1992 Agarwal et al., 1994 and Taj et al., 2016a Duhan et al., 2021
*Alternaria brassicicola*	*AB-toxin*	Otani et al., 1998
*Albugo candida*	*CCG (CxxCxxxxxG)* *CRN* *Ac-RxL*	Furzer et al., 2022 Stam et al., 2013 Links et al., 2011
*Albugo laibachii*	*CCG, RxLR and CRN*	Kemen et al., 2011
*Sclerotinia sclerotiorum*	*SsNEP1 and SsNEP2* *Sscaf-1* *SsSSVP1* *SsCP1* *Six “SsNEs”* *SsITL* *SsCM1* *SsCVNH* *ssv263* 57 effector candidates (11 validated)	Bashi et al., 2010 Xiao et al., 2014 Lyu et al., 2016 Yang et al., 2018 Seifbarghi et al., 2020 Zhu et al., 2013 Djamei et al., 2011 Lyu et al., 2015 Liang et al., 2013 Gupta et al., 2022
*Hyaloperonospora* species (*H. arabidopsidis* and *H. parasitica*)	*HaRxL106* *HaRxLL470* *HaRxL23* *HaRxL17* *HaRxL10* *HaRxL21* *HaRxL77* *HpasRNA*	Wirthmueller et al., 2018 Chen et al., 2021 Deb et al., 2018 Caillaud et al., 2012 Anderson et al., 2019 Harvey et al., 2020 Liu et al., 2022 Dunker et al., 2020

**Table 2:** The R gene(s)/QTLs and the defense-related genes identified in the Brassicaceae family against major fungal pathogens.

Pathogen and disease	Gene (s)/QTLs	Host species	Reference
*Alternaria brassicae*, *Alternaria brassicicola*, *Alternaria raphani*, and *Alternaria alternata*; Narrow-host range necrotroph (NHN) causing leaf blight disease	*BAR and neo* *Hevein* *Chitinase* *Osmotin* *Osmotin-ferritin* *PR-1* *MSRA1* *Lectin* *PR-1, PR-2, PR-3, NPR1* and *PDF1.2* *NPR1* *MPK3* *RtAbeCvG2-1* and *RtAbeCZ5-1* *ARF10 NHL10, HCHIB* and *XLG2* *NAC TFs* *LYK4* 1 and 2 QTLs on two chromosomes (5 and 11 respectively)	*B. napus* *B. juncea* cv. RLM198 *B. juncea* *B. juncea* *B. juncea* cv. Pusa Jaikisan *S. alba* *B. juncea* cv. Varuna *B. juncea* cv. Varuna *B. juncea and S. alba* *B. juncea* cv. Varuna *B. juncea* *A. thaliana* *S. alba* *A. thaliana* *S. alba* *S. alba* *S. alba* + *B. juncea* somatic hybrids	De Block et al., 1989 Kanrar et al., 2002 Mir et al., 2020 Bashir et al., 2015; Munir et al., 2016; Rawat et al., 2017 Taj et al., 2004 Nirupa et al., 2007 Mazumder et al., 2013 Rustagi et al., 2014 Kumar et al., 2015 Nayanakantha et al., 2016 Ali et al., 2017 Tasleem et al., 2017 Rajarammohan et al., 2017 Mukherjee et al., 2019 Pathak et al., 2020 Mondal et al., 2020 De et al., 2021 Singh et al., 2021
*Albugo candida*; oomycete and obligate biotroph causing white rust in brassica	Single dominant resistance gene against *AC-1* *Acr* gene linked with RFLP markers *RAC1*, *RAC2* and *RAC3* *WRR4* *Ac-21* *Ac2* *AcB1-A4.1 and ACB1-a5.1* *ACA1* *ACA1* *Ac2V1* Close relationship of IP markers *At5g41560* and *At2g36360*were established with *AcB1-A4.1 and ACB1-a5.1* *WRR4A*, *WRR4B*, *WRR8*, *WRR9*, and *WRR12* *BjuWRR1* on AcB1-A5.1 *BjuA046215* *PR-5* (thaumatin-like protein-encoding gene) is responsible for enhancing resistance while *CYP20-3* suppressed defense responses *OASTL-B*, *CSD2*, *ACD2*, *MAPK3*, *MAPK6* increased resistance while *MAPK4* and *CYP20-3* enhanced susceptibility Differentially methylated regions	*Raphanus sativus* *B. juncea* *Arabidopsis thaliana* *Arabidopsis thaliana* *B. juncea* *B. juncea* *B. juncea* *B. rapa* *B. napus* *B. napus* *B. juncea* *Arabidopsis thaliana* *B. juncea* *B. juncea* var. Tumida *X B. juncea* var. Varuna *B. juncea* *B. juncea* *B. rapa* subsp. *Perviridis*	Humaydan and Williams, 1976 Cheung et al., 1998 Borhan et al., 2001 Borhan et al., 2008 Prabhu et al., 1998 Varshney et al., 2004 Panjabi-Massand et al., 2010 Kole et al., 1996 Ferreira et al., 1994 Somers et al., 2002 Singh et al., 2015 Cevik et al., 2019 Arora et al., 2019 Bhayana et al., 2020 Kaur et al., 2011a Modak et al., 2022 Tirnaz et al., 2022
*Sclerotinia sclerotiorum*; Broad-host range necrotroph (BHN) causing stem rot	3 QTLs at the seedling stage and 3 at the mature plant stage 9 QTLs in segregating DH populations 21 QTLs from DH population derived from DH821 (Resistant) × DHBao604 (Susceptible) 13 QTLs for stem and leaf resistance 6 and 12 QTLs for stem and leaves resistance 5 and 6 QTLs in controlled conditions while 17 flowering time QTLs linked with SSR resistance 35 QTLs (27 for stem and 8 for leaf resistance) 3 QTLs on C04, C06 and C08 3 QTLs on A08, C06 and C09 10 marker-trait associations for SSR resistance 6 marker loci in A and B genome QTLs for SSR associated with QTLs for flowering time on chromosomes A02, A03, C02 C06 13 significant loci for resistance 3 QTLs for SSR resistance 3 QTLs for 36 candidate genes for SSR resistance	*B. napus* *B. napus* *B. napus* *B. napus* *B. incana* X *B. oleracea* var. *alboglabra* *B. napus* *B. napus* *B. napus* *B. napus* B-genome introgressions from *B. fruticulosa* to *B. juncea* Introgression lines of *B. juncea* X *Erucastrum cardaminoid* *B. napus* *B. juncea X B. fruticulose* introgression lines Bigparental population from *B. napus* var. Zhongshuang 9 X *B. incana* *B. napus*	Zhao and Meng, 2003 Zhao et al., 2006 Yin et al., 2010 Wu et al., 2013 Mei et al., 2013 Wei et al., 2014 Li et al., 2015 Wu et al., 2016 Gyawali et al., 2016 Rana et al., 2017 Rana et al., 2019 Wu et al., 2019 Atri et al., 2019 Mei et al., 2020 Qasim et al., 2020
*Hyaloperonospora parasitica*; oomycete and obligate biotroph causing downy mildew	Single dominant gene Single dominant gene against Race 2 Two Partially dominant genes *Pp523* at the adult stage *Pp523* at chromosome C8 *Pp523* syntenic region at chromosome 1 Partial resistance in the DH population Two dominant unlinked genes at the seedling stage *RPP8* and *RPP9* Two dominant genes at cotyledonary and one at the adult stage A recessive resistance gene *RPP1* *RPP2A/RPP2B* *RPP4* *RPP5* *RPP8* *RPP13* *RPP31* QTL on A08 One SCAR (*SCK14-825*) and two SSR markers (*kbrb006c05-2* and *kbrb058m10-1*) were found to be linked with *BrDW* QTL on chromosome 8 4 major QTLs (*sBrDM8*, *yBrDM8, rBrDM8*, and *hBrDM8*) *BrRHP1* *Ppa3*	*B. napus* *B. oleracea* *B. napus* *B. oleracea* *B. oleracea* *B. oleracea* *B. oleracea* *B. oleracea* *Arabidopsis thaliana* *B. oleracea* *B. oleracea* *Arabidopsis thaliana* *Arabidopsis thaliana* *Arabidopsis thaliana* *Arabidopsis thaliana* *Arabidopsis thaliana* *Arabidopsis thaliana* *Arabidopsis thaliana* *B. rapa* ssp. *pekinensis* *B. rapa* ssp. *pekinensis* *B. rapa* *B. rapa* *B. oleracea*	Lucas et al., 1988 Dickson and Petzoldt, 1993 Nashaat et al., 1997 Coelho et al., 1997 Carlier et al., 2011 Farinhó et al., 2007 Jensen et al., 1999 Wang et al., 2001 Borhan et al., 2001 Monteiro et al., 2005 Carlsson et al., 2004 Botella et al., 1998 Sinapidou et al., 2004 Van Der Biezen et al., 2002 Parker et al., 1997 McDowell et al., 1998 Bittner-Eddy et al., 2000 McDowell et al., 2005 Yu et al., 2009 Yu et al., 2011 Yu et al., 2016 Kim et al., 2011 Singh et al., 2012

**Table 3:** The genes transferred from other species to Brassica members for conferring resistance against major fungal pathogens.

Pathogen	Gene/Protein	Transferred from	Mechanism	Transgenic *Brassica* species	Reference
*Alternaria brassicae*	*Hevein* *Chitinase* *Glucanase* Class II *Chitinase* *PmAMP1* *MSRA1* *Lectin* *NPR1* *MPK3*	*B. juncea* cv. RLM198 *Nicotiana tabacum* *Solanum lycopersicum* *Hordeum vulgare* *Pinus monticola* *B. juncea* cv. Varuna *B. juncea* cv. Varuna *B. juncea* cv. Varuna *B. juncea*	Binding to fungal cell wall carbohydrates (immobilization) Fungal cell wall degradation Fungal cell wall degradation Fungal cell wall degradation Not characterized Membrane antagonist Binding to fungal cell wall carbohydrates (immobilization) Inducing SA pathway Inducing SA pathway	*B. juncea* *B. juncea* *B. juncea* *B. juncea* *B. napus* *B. juncea* *B. juncea* *B. juncea* *B. juncea*	Kanrar et al., 2002 Bashir et al., 2015 Mondal et al., 2007 Chhikara et al., 2012 Verma et al., 2012 Rustagi et al., 2014 Kumar et al., 2015 Ali et al., 2017 Tasleem et al., 2017
*Alternaria brassicicola*	*Endochitinase* gene ‘*ThEn42*’ *Endochitinase* gene ‘*ech42’* Synthetic *chitinase* (*NIC*) *BjLYK4*	*Trichoderma harzianum* *Trichoderma harzianum* - *S. alba*	Fungal cell wall degradation Fungal cell wall degradation Fungal cell wall degradation Induced JA pathway	*Brassica oleracea* var *italica* *Brassica juncea* *B. juncea* *B. juncea*	Mora and Earle, 2001 Kamble et al., 2016 Munir et al., 2016 De et al., 2021
*Albugo candida*	*WRR4*	*Arabidopsis thaliana*	Induced JA/ET pathway	*B. napus*	Borhan et al., 2010
*Sclerotinia sclerotiorum*	*Chitinase* and *Beta-1,3-glucanase* *OXO* *MPK4* *Ovd* *scFv* *Sporamin and chitinase (PjChi-1)* *LTP* *LJAMP2* *MSI-99m* *Bgn13.1* *WRKY33* *Endochitinase42* *Defensin* *PGIP2* *OsPGIP2* *GDSL1* *NPR1* *pBnGH17D7* *LJAMP2* Knocking out *BnF5H*, responsible for lignin pathway	*B. napus* *Triticum aestivum* *B. napus* *Orychophragmus violaceus* *S. sclerotiorum* *Ipomoea batatas and Paecilomyces javanicus* *Oryza sativa* *Leonurus japonicus* *Xenopus laevis* *Trichoderma virens* *B. napus* *Trichoderma atroviridae* *Raphanus sativus* *Phaseolus vulgaris* *Oryza sativa* *Arabidopsis thaliana* *B. napus* *Arabidopsis thaliana* *Leonurus japonicus* CRISPR/Cas9	Fungal cell wall degradation Breakdown of fungal oxalic acid Induced JA pathway Increased the permeability of the fungal membrane Immobilisation and defense activation Protease inhibition and fungal cell wall degradation Membrane antagonist Membrane antagonist Membrane antagonist Fungal cell wall degradation Induction of both SA and JA pathways Fungal cell wall degradation Increase fungal cell membrane permeability Inhibition of fungal endo-polygalacturones Inhibition of fungal endo-polygalacturones Induction of plant defense and phosphatidic acid is released from the fungal cell membrane Induction of SA pathway Host-induced gene silencing Constitutive expression of defense-related genes like PR-1 Decreasing S/G lignin compositional ratio	*B. napus* *B. napus* *B. napus* *B. napus* *B. napus* *B. napus* *B. napus* *B. napus* *B. napus* *B. napus* *B. napus* *B. napus* *B. napus* *B. napus* *B. napus* *B. napus* *B. napus* *B. napus* *B. napus* *B. napus*	Lan et al., 2000 Dong et al., 2008 Wang et al., 2009 Wu et al., 2009 Yajima et al., 2010 Liu et al., 2011 Fan et al., 2013 Jiang et al., 2013 Sang et al., 2013 Kheiri et al., 2014 Wang et al., 2014 Moradyar et al., 2016 Zarinpanjeh et al., 2016 Ziaei et al., 2016 Wang et al., 2018 Ding et al., 2020 Wang et al., 2020 Lin et al., 2022 Jiang et al., 2022 Cao et al., 2022
*Peronospora parasitica*	Catalase E (*Kat E*)	*E. coli*	Hydrogen peroxide dismutation	*B. napus*	El-Awady et al., 2008

## Alternaria leaf blight

Alternaria leaf blight or leaf spot (ALB) is a devastating disease caused by members of the genus *Alternaria*, which is a narrow-host range necrotroph (NHN). Nees von Esenbeck (1816) illustrated the *Alternaria* genus for the first time. The pathogen is cosmopolitan and found across the globe including India (Kadian and Saharan, 1983; Saharan et al, 2016a), Europe (Gladders, 1987), and Canada (Berkenkamp and Kirkham, 1989). The *Alternaria* group contains four species that infect Brassica (Meena et al., 2022). Quarantine testing of exotic rapeseed-mustard germplasm from as many as 20 countries, reported that *A. brassicicola* was more prevalent in seeds as compared to *A. brassicae*. Though *A. raphani* is mainly seen in radish, it may also attack other rapeseed-mustard members (Akhtar et al., 2017). On the other hand, *A. alternata* has a relatively broader host range but is weak as compared to its other counterparts (Verma and Saharan, 1994). Heavy losses ranging from 10% to 70% have been reported due to ALB Saharan et al, 2016a The pathogen severely infects all the above-ground parts of a plant such as pods/siliques, leaves and stems. The leaves give a characteristic target-board symptom caused due to formation of an interrupted necrotic zone (Meena et al., 2016; Jyoti et al., 2021). Grey spots are the characteristic feature of plants infected by *A. brassicae* while *A. brassicicola* causes black velvety sooty spots (**Figures 3A, B**). The hypocotyl of the plant has symptoms of sharp-edged lesions of dark brown colour produced by *A. raphani* (Meena et al., 2010). The environment is an important determinant for disease development as landing, adhesion, penetration, and host colonisation are all dependent on it (Macioszek et al, 2018). In temperate countries, the pathogen survives on plant debris (Humpherson-Jones, 1991), but for sub-tropical or tropical conditions like Indian subcontinent, the survival takes place on unconventional off-season crops, vegetable brassica, and the alternative hosts (Verma and Saharan, 1994; Mehta et al., 2002). The soil-borne and airborne conidia are responsible for secondary spread of the disease (Fatima et al., 2019).

CAZymes (Carbohydrate-active enzymes) are secreted by *Alternaria* for degrading and breaking the host cell wall. When checked at 2 and 4 dpi, 14 Polysaccharide lyases (PLs) were found to be upregulated but polygalacturonases, which cause degradation of pectin by hydrolysing the α-1,4 glycosidic bonds, were found to be upregulated only at 4 dpi (Rajarammohan, 2022). Secondary metabolites/toxins play a huge role in the pathogenicity of *Alternaria*. Around 70 toxins have been reported so far, and out of those 20 are HSTs (Host-specific toxins) (Nishimura and Kohmoto, 1983; Wolpert et al., 2002; Keswani et al., 2016; Taj et al., 2016b). The HSTs interact with the dominant or specific host-susceptibility gene and cause disease and toxicity symptoms. Thus, they are also considered effectors (Friesen et al., 2008). Suzuki et al. (1970) isolated *Destruxin* from *Metarrhizium anisopliae*. *A. brassicae* produces *Destruxin-B*, which is an important HST giving peculiar symptoms of necrosis and chlorosis in the host-specific plant (Meena and Samal, 2019). Another HST produced by *A. brassicae* is the *ABR-toxin* which induces water-soaked lesions followed by chlorosis (Agarwal et al., 1994). *AB-toxin* is a 35 kDa HST produced by *A. brassicicola*. (Otani et al., 1998) The pathogen spores recognise a host-derived oligosaccharide and initiate the production of *AB-toxin* (Oka et al., 2005). Both *ABR* and *AB-toxin* have similar host range and host-specificity, with molecular weight being the only recognisable differential (Meena and Samal, 2019). Most of the HST gene clusters are located on dispensable or supernumerary chromosomes (Akagi et al., 2009).

Rajarammohan et al. (2019a) prepared a contiguous genomic assembly for *A. brassicae* by Nanopore MinION sequencing. Two isolates of *A. alternata*; *PN1* and *PN2* were also sequenced by Rajarammohan et al. (2019b). Potential effector repertoire was predicted for six *Alternaria* species out of which most of them were found to be overlapping and common. It was found that the proportion of effectors is more in the dispensable chromosomes (2.39%) as compared to the core chromosomes (1.69%) (Rajarammohan et al., 2019b). Nep1-like proteins (*NLPs*) are an important class of necrotrophic effectors that induce necrosis and ethylene production. Duhan et al. (2021) identified and characterised two *NLPs* of *A. brassicae*; *AbrNLP1* and *AbrNLP2*. Both the *NLPs* happened to be secretory in nature but differed in their localization in plants. The transcript level of both genes in the initial stages of infection was found to be upregulated, thus hinting at their role in pathogenesis. PTI and necrosis were induced by *AbrNLP2* in both host and non-host species, but the necrosis-inducing ability was absent in *AbrNLP1*. Most of the necrotrophic species of the *Alternaria* genus had two copies of the *NLP* gene but some endophytes possessed only one. Necrotrophs have a relatively less expanded effector repertoire as compared to their biotrophic counterparts (Dong et al., 2012).

Some Brassica species such as *B. napus*, *B. juncea*, and *B. carinata* have been reported to possess inherent resistance. In this regard, *B. rapa* has been reported to be more susceptible as compared to its counterparts (Chadar et al., 2016). The resistance was also reported in the wild relatives such as *Camelina sativa*, *Capsella bursa-pastoris*, *Eruca sativa*, *Diplotaxis catholica*, *D. erucoides*, *Hemicrambe fruticulose* (Singh et al., 2021a). When *A. brassicae* was inoculated on *B. rapa*, 3-butenyl and 4-pentenyl isothiocyanates were found to be released along with sulphides and sesquiterpenes. The catabolism of glucosinolates is established by the release of isothiocyanate and is an important feature of host resistance (Doughty et al., 1996). Camalexin is an indole-derived phytoalexin synthesised by the *MAPK6* signalling cascade and is responsible for imparting resistance to *Camelina sativa*. Gaur et al. (2018) performed molecular docking to find out the interaction of *MKK9* with *MKK1*, *MKK4*, *MKK5*, *MAPK3* and *MAPK6*. Different genes were also identified that play a role in camalexin synthesis. Yadav et al. (2020) did a histopathological, transcriptional, and biochemical study on *B. juncea*, *Sinapis alba* and *C. sativa*. They identified necrosis to occur early in *B. juncea* (1dpi) as compared to *Sinapis alba* (2dpi) and *C. sativa* (3dpi). An enhanced catalase and hydrogen peroxide activity was observed in *Sinapis alba* and *C. sativa.* Two pathogenesis-related genes, *PR-3* and *PR-12* were expressed only in the two wild relatives, hinting towards their role in *Alternaria* resistance. On the other hand, SA-induced genes were found to be highly expressed in *B. juncea*, which explains its antagonistic activity towards necrotrophs. Thus, both camalexin and jasmonic acid are vital for *Alternaria* resistance. Taj et al. (2011) reported that both *MAPK3* and *LOX* interact to start the biosynthesis of JA. One more phytoalexin, sinalexin (sinalbin) produced by *Sinapis alba* was found to induce resistance against ALB.

*B. oleracea* and *B. napus* were transformed in a genotype-independent manner using selectable marker genes such as *bar* or *neo*. *Agrobacterium* strains having *bar* and *neo* genes infected the hypocotyl explants. Cytokinin concentration, water potential, and RH were reduced to avoid vitrification. 30% efficiency was observed in obtaining the transformed shoots. The copy number of chimeric genes varied from one to three in transformants and was confirmed by Southern blotting and genetic analysis (De Block et al., 1989). Mora and Earle (2001) developed ALB resistance in broccoli by transferring an *endochitinase* gene from *Trichoderma* in an *Agrobacterium*-mediated transfer. Kanamycin resistance followed by PCR and southern blotting was done to confirm the presence of genes in transformants. T_0_ Plants (Primary transformants) were found to have as much as 37 times more endochitinase activity than the control. In selfed progeny, it went up to 200 times. On the other hand, when inoculated with *S. sclerotiorum* no substantial difference was observed from the control. *Hevein* is a chitin-binding lectin having an antifungal activity which was analysed in transformed Indian mustard (*B. juncea* cv. RLM-198) against *Alternaria brassicae.* A cDNA encoding *hevein* was transferred and expressed resistance attributes such as small necrotic lesions, longer latent and incubation period, reduced disease intensity and delayed senescence (Kanrar et al., 2002). Glucan is an important component of the fungal cell wall and is hydrolysed by PR-protein *glucanase*. Mondal et al. (2007) used the CaMV 35S promoter to express the class I basic glucanase gene. A stable integration was confirmed by northern and southern hybridisation. Transgenics inhibited *Alternaria brassicae* hyphal growth by 15-54% and heavily delayed the disease incidence as compared to untransformed. Thus, the experiment showcased the efficiency of heterologous PR genes having the ability to impart ALB resistance. Taj et al. (2004) reported that *osmotin* provides resistance against the ALB disease by influencing the cell cycle and cell death pathways. *B. juncea* calli was taken as a model for investigating this phenomenon and *P53* and caspase-like proteins were found to be overexpressed and CDC and cyclin B were suppressed. Finally, they concluded that osmotin is not able to provide resistance but delays the onset of symptoms. *B. juncea* cv. Pusa Jaikisan was transformed by *Agrobacterium* strain *GV2260* containing binary vector *p35SGUSINT* and optimum conditions for transformation were identified based on transient GUS expression. The pre-culture period, age of the explant, bacterial density and silver nitrate were optimised for transformation. PCR and southern and western blotting were done to confirm the presence of transformants in T_0_ and T_1_ (Nirupa et al., 2007). Chhikara et al. (2012) developed resistance by coexpressing type I ribosome-inactivating protein and barley antifungal gene class II *chitinase* through *Agrobacterium*-mediated transfer. Transgenic plants showed a Mendelian inheritance pattern (3:1) and 44% protection was observed in them. Also, a reduction in lesion size, number and expansion, and delay of onset of symptoms was observed in transformed lines.

*PmAMP1* is an antimicrobial peptide isolated from *Pinus monticola* and was able to provide protection to oilseed-brassica against many fungal genera such as *Leptosphaeria maculans*, *A. brassicae* and *S. sclerotiorum*. Higher resistance was observed *in planta* when the cDNA encoding gene was transferred to the *B. napus* genome. Even the *in vitro* extracted proteins were observed to have antifungal activity (Verma et al., 2012). Mazumder et al. (2013) observed a peculiar difference in disease response given by the host (*B. juncea*) and non-hosts (*Sinapis alba* and *Arabidopsis thaliana*) against *A. brassicicola*, where the former activates the SA pathway and JA/ABA is induced in the latter ones. This explains that the pathogen modulates the plant defense response in favour of biotrophic mode in host species whereas it is unable to do so in non-hosts as they can counter it by the JA pathway. Broad spectrum resistance against fungal necrotrophs is imparted by cationic antimicrobial peptides (CAPs). Many synthetic compounds have been derived from these proteins. *MsrA1* is a chimeric protein formed by the combination of melittin CAPs and cecropin A. Five transgenic lines were checked for resistance against ALB and SSR. Up to 71.5% and 85% protection were observed for SSR and ALB, respectively in transgenic *B. juncea* (Rustagi et al., 2014). A *chitinase* gene from *Streptomyces griseus* was used to transform cotyledons and hypocotyls. Hypocotyls were reported to be more responsive, and 2 mg/L BAP and 0.2 mg/L were the best hormonal combination for callus transformation. Transgenic callus was confirmed by PCR (Bashir et al., 2015). Kumar et al. (2015) transformed *B. juncea* using chickpea *lectin*. *Lectin* is a vital secondary metabolite present everywhere and binds reversibly and specifically to carbohydrates, providing resistance against biotic stresses such as ALB and abiotic stresses such as drought and salinity. As much as 60% protection was observed in the transformants and higher proline content and augmented water retention capacity was also seen. *CaMV 35S* promoter was used in an *Agrobacterium tumefaciens*-mediated (EHA101) transfer having synthetic *chitinase* gene (*NIC*) in *pEKB/NIC*. Considerable resistance was seen against *A. brassicicola* and, the presence of *chitinase* gene in transformants was validated by PCR and southern hybridisation (Munir et al., 2016). Another *endochitinase* gene ‘*ech42*’ was transferred from *Trichoderma virens* to *B. juncea* and up to 73% protection was seen against both *A. brassicae* and *A. brassicicola*. The presence of the transgene was confirmed by a fluorimetric zymogram (Kamble et al., 2016).

Nayanakantha et al. (2016) comparatively analysed the expression of defense-related genes in *Sinapis alba* and *B. juncea* and reported all 5 genes, viz. *PR-1*, *PR-2*, *PR-3*, *NPR-1* and Plant *Defensin 1.2* (*PDF1.2*) to be highly expressed in *S. alba* as compared to Indian mustard; when inoculated with *A. brassicae*. *PDF1.2* is a jasmonic acid-induced gene and was shown to have higher transcript levels in *A. thaliana* against the necrotrophs. *PR-1* on the other hand was induced by the SA pathway. But, in this case, both SA and JA-induced genes were expressed earlier and more in *Sinapis alba* than in *B. juncea*. This confirmed that the response varied from Indian mustard to *A. thaliana*. An expression analysis experiment was conducted to characterise *B. juncea* class IV *chitinase* against JA, SA, wounding and *Alternaria* infection. A *chitinase* promoter of size 2.5 kb was isolated and *in-silico* analysis was done further. Finally, it was fused with the *GUS* gene and introduced into *A. thaliana* and, it reportedly showed higher activity after wounding and JA treatment but lower in SA treatment. Organ specificity was analysed based on GUS activity in seeds, siliques, leaves and meristematic cells (Rawat et al., 2017). Ali et al. (2017) isolated and characterised SA-receptor gene *BJNPR1* to play an important role in conferring broad-spectrum resistance against ALB and powdery mildew. No phenotypic abnormalities were observed, and the gene was found to be constitutively expressed. The gene was upregulated on fungal attack or SA treatment, but similar trends were not observed upon ABA and JA treatment. Studies on *MAPK3*, *MAPK4* and *MAPK6* in *A. thaliana* have indicated that, they are involved in conferring resistance against multiple biotic stresses. Tasleem et al. (2017) checked the expression of these genes in transgenic *B. juncea* (over expressed *MAPK3*). *MAPK3* and *MAPK6* were expressed in the early stages of *Alternaria* infection whereas *MAPK4* was activated in the later part. This showed the SA pathway to be an important determinant of ALB resistance and *MAPK3* interacts with it in a positive manner. *Arabidopsis* can be used for mapping the QTLs for ALB resistance and identifying paralogues in *Brassica*. In this regard, Rajarammohan et al. (2017) developed three biparental mapping populations with the help of two susceptible lines (Gre-0 and Zdr-1) and three resistant lines (CIBC-5, Ei-2 and Cvi-0). Six QTLs were identified, out of which five were population-specific and one was common to all accessions. 50% of the variation was conferred by two QTLs which had a larger effect, and resistance was confirmed to be quantitative in nature as both population-specific and common QTLs were found. ABA-auxin cross talk is less studied as compared to JA-SA.

Mukherjee et al. (2019) reported an enhanced expression of the auxin-responsive factor *ARF10* along with, the expression of *ARF16* and *ARF17*. When *ARF10* was expressed in transgenic *Brassica juncea*, it led to ABA sensitivity and increased resistance against *A. brassicicola*. Many ABA-responsive genes like *ABI3*, *ABI4* and *ABI5* were also induced without having any profound effect on auxin-biosynthesis. The *ARF10* interacted with the promoter of *ABI5* and conferred ABA sensitivity, finally culminating in a defense response. *HCHIB* and *NHL10* were identified as major defense-related genes against ALB in *Arabidopsis*. Some genes such as *WRKY*, *CZF1*, *MP*, *AXR3*, *IAA1*, *IAA19*, *ARF6*, and *XLG2* modulated the JA, SA, and ET pathways. *XLG2* showed a more elevated response against *Alternaria brassicicola*. Similar genes were also found and characterised in the *B. rapa* genome by using *Arabidopsis* as a model plant system (Pathak et al., 2020). Mir et al. (2020) evaluated *C. sativa* and *B. juncea* for chitinase genes and reported 79 and 47 of them respectively. The expression of these chitinase gene was confirmed by qRT-PCR which was found relatively more in *C. sativa* confirming its comparative tolerance. *NAC TFs* are a specific class of transcription factors which impart resistance against multiple abiotic and biotic factors. The *NAC TFs* were comparatively analysed in *S. alba* (resistant) and *B. juncea* (susceptible). Out of thirteen selected NAC TFs, six were found to be highly expressed in treated and tolerant *B. juncea* and *S. alba*. The NACs were instrumental in resistance against both wounding (abiotic stress) and *Alternaria* inoculation (Mondal et al., 2020). Plant defense response is activated by the Lysin motif receptor-like kinases (*LYKs*) by recognising chitin and De et al. (2021) reported it to be strongly induced in *S. alba* as compared to *B. juncea*. Though *B. juncea* had the *LYK4* domain, it lacked many key protein kinases and was found to be inactive. ALB resistance was gained by overexpressing the *BjLYK4* gene and many JA-induced genes and chitin-responding *WRKY* transcription factors were also highly expressed upon pathogen attack. Singh et al. (2021b) introgressed QTLs from *S. alba* to the backcross progenies of *S. alba* and *B. juncea* somatic hybrids which were more stable. The quantitative nature of resistance was confirmed by differential response seen in the backcross population ranging from highly resistant to susceptible. One and two QTLs were detected by ICIM-ADD mapping in chromosomes 5 and 11 respectively. 5.51-10.87% variation was observed for disease resistance in the backcross population. Before this, the QTL introgression for Alternaria leaf blight has never taken place from a brassica family member to cultivated rapeseed-mustard.

## White rust

*Albugo candida* is an obligate biotroph (Kaur et al., 2011b) belonging to the class oomycetes, causing the dreaded white rust (WR) disease in all the major oilseed brassica (OSB) growing countries such as India (Chowdhary, 1944; Kolte, 1985), Pakistan (Perwaiz et al., 1969) Canada (Petrie, 1973), Germany (Klemm, 1938), Japan (Hirata, 1954), South Korea (Choi et al., 2007), China (Zhang et al., 1984), New Zealand (Hammett, 1969), United Kingdom (Berkeley, 1848), and USA (Walker, 1957). Petrie and Vanterpool (1974) reported that systemic infection of white rust can lead to a 60% loss in seed yield. In Indian conditions, 60% yield losses are reported due to the combined effect of leaf and inflorescence infection (Lakra and Saharan, 1989). Bal and Kumar (2014) reported average 36.88% yield loss due to white rust disease. As white rust is an obligate biotroph, signs are more pronounced as compared to symptoms. The white blister-like pustules starting from the underside of foliage and spreading to the foliar parts are characteristic symptoms of the disease (**Figures 3E, F**). Further, the systemic infection leads to the formation of the staghead phase (SP), which is a combined effect of hypertrophy and hyperplasia (Verma and Petrie, 1980; Kolte, 1985) (**Figure 3E**). Sporangiophore produces basipetal chains of sporangia between the epidermis and mesophyll layer of host tissue. Direct or indirect germination by zoospores takes place after the release of sporangia (Xiao et al., 2022). Finally, intercellular hyphae penetrate the host cell and sporangia and oospores are established as resting structures (Meena et al., 2014a). *A. candida* has a wide host range and can cause infection in around 63 genera and 241 host species (Gupta and Saharan, 2002; Choi et al., 2007; Mishra et al., 2009; Saharan et al., 2014, Pound G.S. and Williams, 1963 isolated pathogen from various host species and identified 6 races of *A. candida*. Singh et al. (2021) collected and characterised 13 isolates of *A. candida* and 1 isolate of *Wilsonia bliti* based on their morphological features and the highly conserved COX2 and ITS data.

*Albugo candida* has a relatively smaller genome (45.3 Mb) and less abundance of pathogenicity-related proteins encoding genes like *RxLR* effector, *Elicitins* and *CRINKLER*-like genes as compared to other biotrophic oomycetes like *Hyaloperonospora arabidopsidis* (99Mb). Still, 26 *Ac-RxLs* were identified in the pathogen effector repertoire and despite possessing a degenerate *RxLR-dEER* motif, it was able to cause death in host cells (Links et al., 2011). An improved reference genome was prepared by Furzer et al. (2022) which led to a 175% expansion in the candidate repertoire of *CCG* class effectors. Albuginales have pathogenetically evolved separately from the Peronosporales, which are characterised by having large families of *CRN* and *RxLR* effectors (Haas et al., 2009; Baxter et al., 2010). 13 similar proteins to *CRN* were identified in the secretome of *Albugo candida*. (Stam et al., 2013). Their presence and expansion indicate a common *CRN* present before acquiring pathogenicity and their possible role in parasitising the host. Castel et al. (2021) found out that the *col-0* (Columbia) accession of *Arabidopsis thaliana* has three TIR-NLR encoded genes at *White Rust Resistance 4* locus (*WRR4*). The *CCG* effectors of *A. candida races* 2, 4, 7 and 9 (from *B. juncea*, *Capsella bursa-pastoris*, *B. rapa* and *B. oleracea*) were recognised by the *col-0* alleles of *WRR4A* and *WRR4B*. On further analysis, it was concluded that four *CCG* effectors were recognised by *WRR4B* and eight of them interacted with *WRR4A*. Thus, *WRR4* paralog-based broad-spectrum resistance for multiple *A. candida* races was established. This resistance was found to be broken by an isolate *AcEx1*. A *WRR4A* allele; *WRR4A^Oy-0^* in Arabidopsis accession was identified and found to confer resistance against the *AcEx1*, but it substantially reduced the recognition of *WRR4A*-recognised effectors (Castel et al., 2021). Races 2 (non-host) and 7 (host) were intercrossed by Adhikari et al. (2003) and a ratio of three avirulent to one virulent was obtained upon inoculating F_2_ population of *B. rapa*. This evidently proves a resistance-gene mediated recognition of the *Ac2V* effector and may hint towards the genetic basis of non-host resistance. The mechanism of NHR in *Brassica* species is vital for resistance breeding as new effector repertoire can evolve due to genetic exchanges between races of *A. candida* (McMullan et al., 2015). The recognition of effectors by the non-host intracellular receptors may indicate a common ancestral host species for the pathogen (Lee et al., 2014; Cevik et al., 2019).

The white rust resistance gene for race 1 of *Albugo candida* (AC1) in *R. sativus* was studied along with six other characters. The pink pigmentation (pi) in plants was found to be closely linked with the *AC1* gene (Humaydan and Williams, 1976). A restriction fragment length polymorphism marker (RFLP) was identified in close association with the white rust resistance gene (*Acr*) in *B. juncea* (Cheung et al., 1998). Panjabi-Massand et al. (2010) selected two east European lines, Heera and Donskaja-IV and tagged two independent loci governing white rust resistance. They acquired two doubled haploid populations by crossing Heera with Varuna and TM4 with Donskaja-IV. After crossing, a single major locus was identified in both cases, *AcB1-A4.1* was mapped on linkage group A4 in Heera while in Donskaja-IV, the linkage group was *AcB1-A5.1* on A5. Synteny between *Arabidopsis* and *B. juncea* formed the basis for developing closely flanked markers and introgression of the resistant loci is easier with these markers. Singh et al. (2015) checked the genotype non-specific intron polymorphic (IP) markers *At5g41560* and *At2g36360* from *Arabidopsis* and concluded their proximity with loci responsible for white rust resistance *AcB1*-*A4*.*1* and *AcB1-A5.1*, respectively in *B. juncea*. Two accessions were selected from *A. thaliana*, Ksk-1 and Ksk-2 and three white rust resistance genes (*RAC1*, *RAC2* and *RAC3*) belonging to class TIR-NB-LRR were mapped and *RAC1* was cloned (Borhan et al., 2001). Another *R* gene, *WRR4* belonging to class TIR-NB-LRR was characterised by Borhan et al. (2008). This gene was identified as the basis of broad-spectrum resistance conferred against four races of *A. candida*. Borhan et al. (2010) suppressed the enhanced disease susceptibility-1 (*eds*-*1*) gene through RNA interference, and they transferred the *WRR4* gene and expressed it in the susceptible lines of *B. napus* and *B. juncea* to get resistance against race 2 and 7 of *A. candida*.

Prabhu et al. (1998) crossed resistant and susceptible varieties of *B. juncea* to obtain the doubled-haploid population derived from F_1_. They identified two markers *WR2* and *WR3* which determined the presence or absence of the white rust resistance gene, *AC21*. A white rust resistance locus, *AC2(t)* was mapped with the help of the RAPD marker by Varshney et al. (2004) and they also used bulk segregant analysis and AFLP to develop a more tightly linked marker. Two cultivars of *B. napus* were crossed (Major and Stellar) and a backcross population (BCP^2^), F^1^-derived doubled haploid and F^2^ population were analysed to identify a single dominant locus for white rust resistance, *ACA1* on linkage group 9 (Ferreira et al., 1994). A similar experiment was carried out on *B. rapa* by Kole et al. (1996) in which they crossed a resistant (Per) and susceptible (R-500) cultivar. They mapped the *ACA1* locus on linkage group 4 with 144 RFLP loci segregating in the F_3_ generation. The *ACA1* locus was 13.3 cM apart from the leaf pubescence locus (*PUB1*), so both *PUB1* and RFLP markers can be used for the introgression of *ACA1* (Kole et al., 1996). For transferring the canola quality, an interspecific cross has been made between *B. napus* and *B. juncea*. This has facilitated the introgression of a white rust resistance gene from *B. napus* to *B. juncea*. A BC_3_F_2_ population of *B. juncea* was used to identify DNA markers for the white rust resistance trait. Eight *B. napus*-derived AFLP markers along with white rust resistance gene (*AC2V1*) were identified (Somers et al., 2002). *R*-gene-mediated non-host resistance (NHR) can be instrumental in providing durable resistance to crop varieties so that they can be transferred from source to the susceptible plants. The adult plants of *Arabidopsis* were found to be resistant to white rust disease. 593 inbred lines developed from the *Arabidopsis* MAGIC population were screened and two susceptible transgressive segregants were identified. One of four genes was speculated to be the basis of resistance against the *AC2V* isolate of *A. candida*. An additional gene was identified for race 9 infecting the *B. oleracea* crop (Cevik et al., 2019).

Arora et al. (2019) identified and functionally characterised a CC-NB-LRR protein-encoding gene, *BjuWRR1*. This constitutively expressed gene was responsible for conferring broad-spectrum complete resistance against white rust in Donskaja-IV. *BjuWRR1* located on *AcB1-A5.1* was introgressed in Varuna, Rohini, Pusa Jaikisan and Pusa Bold. The developed NILs were found to be resistant against all the six isolates (Pantnagar, Meerut, Bharatpur, Samastipur, Hisar and Alwar) tested. The allele-specific molecular markers can be used for transferring the locus into new lines and developing hybrids. A cross was performed between *B. juncea* var. Tumida and *B. juncea* var. Varuna and the F_1_DH population were used to map a new R-gene locus for isolate *ACB1* which is located on LGA6 of *B. juncea* var. Tumida. The candidate gene, *Bju*A046215 is a CC-NB-LRR and its alleles in susceptible varieties produce a truncated LRR-domain protein. Both *BjuWRR1* and *BjuA046215* belonged to the *CNL-D* group of *R* genes, and they were phylogenetically similar (Bhayana et al., 2020). DNA methylation as a determinant of epigenetic resistance plays an important role in plant immunity. Its role in *Brassica* has not been characterised yet. Tirnaz et al. (2022) evaluated *B. rapa* subsp. *perviridis* for modification in its whole genome DNA methylation against white rust. “Misugi” (susceptible) and “Nanane” (resistant) were analysed for differentially methylated regions (DMR) and 233 and 275 DMRs were identified respectively. DMRs were located within genes in “Mishugi”, while they were found to be either upstream or downstream in “Nanane”. This study points towards a potential role of DNA methylation in white rust resistance.

Kaur et al. (2011a) carried out a comprehensive proteomic analysis to find the role of defense compounds and genes in imparting white rust resistance. A total of nineteen proteins showcased a reproducible difference in resistant (CBJ001) and susceptible (RH819) cultivars upon inoculation with *A. candida*. Q-TOF MS/MS was used to confirm the identity of proteins and out of these, five were only reported in the resistant cultivar. *PR-5*, which encoded a thaumatin-like protein, earlier not associated with defense response was found to play role in white rust resistance. *CYP20-3* is an isoform of peptidyl-prolyl cis/trans isomerase (PPIase) and was only identified in the susceptible cultivar, establishing its role in susceptibility. Expression analysis of defense-related genes and *MAPKs* cascade was checked in wild and transgenic Varuna (overexpressing *MAPK3*). The transgenic Varuna was found to be relatively more tolerant as confirmed by disease indexing. *MAPK6* was found to mimic the *MAPK3* pathway and suppress the expression of *MAPK4* in transgenic Varuna. *WRKY29* and *WRKY33* were also reported to be highly expressed in the transformed one. Similarly, transcripts of *OASTL-B*, *ACD2* and *CSD2* also increased, and the *CYP20-3* had a reverse trend as its transcript accumulated more in non-transgenic Varuna. *In-silico* studies were done to study protein-protein interaction, secondary & tertiary structures and finally predict putative phosphorylation sites (Modak et al., 2022).

## Sclerotinia stem rot

Sclerotinia stem rot (SSR), caused by *Sclerotinia sclerotiorum*, is a serious production-impeding factor for rapeseed-mustard. The pathogen attacks a plethora of host species and is thus characterised as a broad-host range necrotroph (BHN). The disease resistance is difficult to develop as host specialisation is rare and around 600 plant species are reportedly infected by it (Liang and Rollins, 2018). Though it is rare, host specialisation does occur and was conclusively proven by Kull et al. (2004). There has been a striking preference for dicots against monocots which can be ascribed to dicots possessing a peculiar form of glycosylinositol phosphorylceramide (Lenarčič et al., 2017) and monocots producing germins against the pathogen (Davidson et al., 2009). Libert (1837) described it for the first time and named it *Peziza sclerotiorum*. Later, Purdy (1979) established the name *S. sclerotiorum* (Lib.) de Bary. Cool and moist weather conditions have proven to be conducive for SSR epiphytotics causing 100% losses (Ghasolia et al., 2004, McDonald and Boland, 2004, Shukla, 2005; Singh et al., 2008). It has been established as one of the major yield-limiting factors of *B. napus* in India, China, Europe, Australia, and Canada (Rana et al., 2017). It was considered a minor disease in India 20 years back, but irrigation facilities and monocropping have made it emerge as one of the major yield-limiting factors in rapeseed-mustard (Sharma et al., 2015). The disease is characterised by white, fluffy mycelia seen on siliquae, stem and leaves (Christias and Lockwood, 1973). Though *Sclerotinia* infects all parts of the plant, the stem is most severely affected which leads to lodging and girdling causing yield reduction (Melouk et al., 1989; Uloth et al., 2016) (**Figures 3C, D**). Sclerotinia showcases both myceliogenic (soil-borne infection) and carpogenic (air-borne infection) germination which leads to symptoms on stem and siliquae, respectively (Singh et al., 2021).

Being a necrotroph; although recent studies point towards a short initial biotrophic phase, the pathogen is thought to employ brute force for damaging the host, but it has some sophisticated mechanisms up its sleeve (Saharan et al., 2017). Sharma et al. (2018) conducted virulence and phylogenetic analysis for 65 isolates of *S. sclerotiorum* utilising morphological features, SSR profiling, and ITS sequencing, and finally established three evolutionary lineages. Oxalic acid was proven to be an important pathogenicity determinant with the help of UV-induced mutations (Godoy et al., 1990; Dutton and Evans, 1996). Later studies have concluded that, rather than directly affecting host physiology, pH manipulation is the main job of oxalic acid (Liang et al., 2015; Xu et al., 2015; Jingtao et al., 2018). Several putative effectors have also been identified in *S. sclerotiorum*. Cell death is vital for a necrotroph to obtain nutrients and in this regard, two *NLPs*; *SsNEP1* and *SsNEP2* were the first cell death-inducing effectors to be identified in tobacco leaves and shown by *Agrobacterium tumefaciens*-mediated expression (Bashi et al., 2010). Xiao et al. (2014) concluded that a gene named, *Sscaf-1* led to the development of sclerotia and compound appressoria formation. Its protein contains a Calcium ion-binding EF-hand motif. Disruption of *Sscaf-1* was done by the transfer DNA insertion and, further knockdown and gene complementation established it as the cause of changes observed in Sunf-MT6. *SsSSVP1* is a small cysteine-rich protein that has the capacity to induce cell death and a reduction in virulence was observed upon silencing the gene. It is a potent plant energy metabolism manipulator as it interacts with and disturbs the subcellular localisation of *QCR8* (Lyu et al., 2016). A cerato-platanin protein (CP), *SsCP1* was characterised, and an accumulation of transcripts occurred during initial stages of infection. It facilitated infection by interacting with *PR1* in apoplast. In transgenic plants possessing SsCP1 gene, an increased level of SA was observed along with broad-spectrum resistance to pathogens such as *Botrytis cinerea*, *Alternaria brassicicola* and *Golovinomyces orontii* (Yang et al., 2018). Six novel effectors; *SsNEs* were discovered by Seifbarghi et al. (2020). A reduction but not nullification in virulence was observed in *SsNE2* when cysteine was substituted by alanine.

Besides inducing necrosis, the *S. sclerotiorum* effectors also target the host defense response. One such effector, *SsITL* was found to be upregulated at 1.5-3 hpi and on further analysis, it was seen that upon inoculating the silenced transformant the defense-related genes such as *PR1* and *PDF 1.2* produced their highest transcript level at 3 hpi which was 9 hours before the response observed for wild strain. It also inhibited the jasmonic acid/ethylene (JA/ET) pathway which is crucial for resistance against necrotrophs (Zhu et al., 2013). A further study by Tang et al. (2020) reported that *SsITL* interacts with the Calcium-sensing receptor (CAS) located in the chloroplast. This interaction inhibited the Calcium ion signalling and finally, the salicylic acid (SA) pathway was inhibited in the early stages of infection. This interaction is essential for virulence as *SsITLs*, which lost their ability to interact with CAS, were unable to infect the host. *SsCM1*, a putative effector shares structural similarities to *Cmu1*, an *Ustilago maydis* effector which also targets the SA accumulation (Djamei et al., 2011; Kabbage et al., 2013). *SsCVNH* is an effector that contains *CVNH* carbohydrate-binding domain and was conclusively found to be upregulated during infection and played an important role in virulence, growth, and formation of sclerotia. The *SsCVNH* is thought to either protect the fungal cell wall or evade PTI response of the host like other fungal effectors having LysM domain (Lyu et al., 2015; Sánchez-Vallet et al., 2020). The *ssv263* is an identified orthologue of *B. cinerea* protein and putatively reported to inflict symptoms in *B. napus*, though the mechanism remains unclear (Liang et al., 2013). Gupta et al. (2022) did a whole genome-sequencing of a highly virulent isolate of *S. sclerotiorum*, “ESR-01” which yielded 57 candidate effectors, out of which 30 were reported to be novel. Expression profiling of the isolate validated 11 of the effector candidates. Metabolisation of the host defense compounds is an effective strategy employed by *S. sclerotiorum* and it also plays a significant role in determining its host range. Similarly, cross-kingdom RNAi transfer from pathogen to host is also seen as a potential area of research in the upcoming future (Derbyshire et al., 2022).

Map-maker QTL was used to detect six QTLs for *S. sclerotiorum* resistance in *B. napus*. Out of these, three were identified at the seedling stage while three were at the adult plant stage. Additive epistatic interactions were also reported and they, along with single locus QTL were responsible for SSR resistance (Zhao and Meng, 2003). Nine QTLs were identified at A2, A3, A5, C2, C4, C6 and C9 for SSR resistance in *B. napus* from two segregating DH lines (HUA population) developed by crossing Chinese and European spring lines (Zhao et al., 2006). Advanced molecular tools were used to map the QTLs or putative *R* genes for SSR resistance. *B. juncea* was crossed with *B. fruticulose* to produce fertile introgression lines with B-genome chromosomes being terminally introgressed. Microsatellite markers were used for genotyping these resistant lines. Association mapping was done to identify ten significant marker-trait associations (Rana et al., 2017). 96 sets of *B. juncea*–*Erucastrum cardaminoid* introgression lines were developed by Rana et al. (2019). Genotyping was done by both transferrable microsatellite markers and sequencing to confirm marker-trait associations. SSR markers were used to identify the association between resistance and six marker loci in A and B genomes. Based on GWAS analysis, SNP markers were characterised to be linked to SSR resistance in B03, A06 and A03 chromosomes.

A plethora of resistance mechanisms was identified by annotation studies such as production of anti-fungal metabolites, hypersensitive response and signal transduction. The *LRR-RLK* genes were found to be associated with total five *SNPs* on A03 chromosome and genetic factors for both PTI and ETI were found on A03. Three *R* genes encoding TIR-NBS-LRR was identified, but till now no cloning has been done for any *R* gene against SSR (Wu et al., 2009; Wu et al., 2013). The susceptible *B. oleracea* var. *alboglabra* was crossed with resistant wild *B. oleracea* (*B. incana*) to give a biparental population exhibiting 6 and 12 QTLs for stem and leaf resistance, respectively. Two QTLs were reportedly identified on C09 for both leaf and stem resistance. With the help of blasting to *B. rapa* as a reference genome, it was found that chromosome C09 harbours the candidate *R* gene. About 30 genes were identified at 2.7 Mb genomic region of A09 as being involved in defense response and resistance-related functions. The putative genes were characterised as *CC-NS-LRR* (Mei et al., 2013). Yin et al. (2010) identified a total of 21 QTLs on A3, A4, C1, C2 and C7 for SSR by evaluating the DH population obtained by crossing *B. napus* cultivars DHBao604 (susceptible) and DH821 (resistant). Putative QTLs have been identified in *B. napus* for SSR resistance. Three candidate QTLs involved in SSR resistance have been identified with the help of GWAS on C04, C06, and C08 (Wu et al., 2016) and A08, C06, and C09 (Gyawali et al., 2016). Few more QTLs on chromosomes A02, A03, C02 and C06 in *B. napus* were identified for SSR resistance by the SNP-array genotyping. The flowering time QTLs were also located in these regions harbouring SSR resistance QTLs (Wu et al., 2019). Introgression lines were prepared from a cross between *B. juncea* and *B. fruticulosa* and were analysed for resistance. A total of 13 loci were found to be significant and the annotation experiment provided 20 candidate genes belonging to defense families such as *Chitinase*, Malectin/receptor-like protein kinase defensin-like (*DEFL*), desulfoglucosinolate sulfotransferase protein and *lipoxygenase* (Atri et al., 2019). Mei et al. (2020) pyramided three SSR resistance QTLs located on C01, C09-1, and C09-2 chromosomes by developing BC1F8 population of cross between *B. napus* var. Zhongshuang 9 and *B. incana*.

Wu et al. (2013) identified a candidate *R* gene *BnaC.IGMT5.a* in *B. napus* based on the differential expression pattern shown by two parental lines. One out of thirteen identified QTLs for SSR was found to be located on C06. Chromosomes A9 and C6 were found to be harbouring the 8-leaf and 27-stem resistance QTLs. This hints towards some of the genotypes sharing common leaf and stem resistance regions (Li et al., 2015). Seventeen QTLs based on the DH population derived from a cross between European winter and Chinese semi-winter for the flowering time trait were found to be in close association with the SSR QTLs on C02 and LG A02 and five and six QTLs were identified in controlled and field conditions, respectively. Common resistance genes were found on both chromosomes A and C pointing towards common chromosome ancestry and SSR resistance being specific at the subpopulation level (Wei et al., 2014). Qasim et al. (2020) used SNP markers to identify 17 QTLs for SSR resistance over three different seasons. No common QTL was identified across all three seasons but three QTLs, *SRA9a*, *SRC2a* and *SRC3a* appeared in two seasons. Stem width was identified as having weak relationship with the resistance trait. Flowering time shared a very strong negative correlation with SSR resistance as the early maturing varieties were found to be more susceptible as compared to the late maturing ones.

Lan et al. (2000) transformed a good-yielding *B. napus* variety H165 by constitutively expressing the *chitinase* and *beta-1,3-glucanase* genes transferred through an *Agrobacterium*-mediated transfer of expression vector *pBLGC*. Oxalic acid is an important determinant for pathogenicity and oxalate oxidase (*OXO*) can oxidise this compound into CO_2_ and H_2_O_2_. A wheat *OXO* gene was constitutively expressed in transgenic rape, and it imparted a considerable amount of disease resistance against SSR which accounted for as high as a 90.2% reduction (Dong et al., 2008). *MPK4* is known to enhance the jasmonic acid-activated defense response which is evident from an experiment conducted by Wang et al. (2009) in which they developed transgenic *B. napus* by overexpressing *BnMPK4* gene that yielded disease resistance against broad-host range necrotrophs such as *S. sclerotiorum* and *Botrytis cinerea*. *B. napus* was transformed by *Agrobacterium*-mediated transfer of a plant defensin gene *Ovd*, that was cloned from *Orychophragmus violaceus*. The RT-PCR analysis confirmed that the expression of *Ovd* was much lower in antisense and non-transgenic plants as compared to the sense lines. 20% reduction in lesion size was reported and the gene was confirmed to confer a strong defense against the SSR disease (Wu et al., 2009). Yajima et al. (2010) transferred a recombinant pathogen-specific antibody (*scFv*) to *B. napus* lines and reported enhanced tolerance. *Brassica napus* var. ZS758 was transformed by a binary vector harbouring sporamin and chitinase *PjChi-1* gene. The transformed plants showcased increased resistance against *Plutella xylostella* and *S. sclerotiorum* (Liu et al., 2011). Anti-fungal and anit-bacterial activities have been reported due to lipid transfer protein (LTP) and, the *Agrobacterium*-mediated transfer of this protein into *B. napus* enhanced the peroxidase (POD) and superoxide dismutase (SOD) activities along with providing tolerance to the transgenic against SSR (Fan et al., 2013). Jiang et al. (2013) employed *Agrobacterium tumefaciens* to transfer a non-specific lipid transfer protein-like antimicrobial protein gene (*LJAMP2*) from *Leonurus japonicus* into *B. napus* genome. It was uniformly transcribed in all transgenics as confirmed by the RT-PCR analysis.

The defense response was initiated in transgenics with an increase in H_2_O_2_ and *PR-1* gene but *PDF 1.2* remained comparatively inactive. Tolerance to SSR was seen in *B. napus* transformed with the *MSI-99m* gene which belongs to the magainins class of antimicrobial peptides and is responsible for broad-spectrum resistance against bacteria and fungi (Sang et al., 2013). Kheiri et al. (2014) transferred a β-1,3-glucanase (*bgn13.1*) gene cloned in a *pUC19* cloning vector from *Trichoderma virens*-10. The transgenic rape showed a high quantum of resistance. Various studies have repeatedly emphasised the role of *WRKY* transcription factors in plant defense. Transformed plants that overexpressed the *BnWRKY33* gene (*WRKY* gene isolated from *B. napus*) enhanced the accumulation of H_2_O_2_ along with increased transcription of *PR-1* and *PDF 1.2* genes (Wang et al., 2014). Moradyar et al. (2016) concluded that along with SP-DDE synthetic promoter, the *SP-DDE* controlled expression of the chimeric *chitinase* gene was also responsible for inhibiting fungal growth and development. The chimeric *Chit42* gene from *Trichoderma atroviridae* and a defensin gene from *Raphanus sativus* along with a *Serratia marcescens* C-terminal fused Chitin binding domain, were transferred and coexpressed in *B. napus*. The results shown were positive as a heterologous source can be used to transfer and induce resistance for SSR (Zarinpanjeh et al., 2016). Ziaei et al. (2016) developed SSR resistance by transferring the *Chit42* gene along with the chitin-binding domain and polygalacturonase-inhibiting protein 2 (*PGIP2*) of *Phaseolus vulgaris*. Another *PGIP* protein was transferred to confer SSR resistance in rape. The *Oryza sativa* gene (*OsPGIP2*) was constitutively expressed in transgenic plants as confirmed by RT-PCR (Wang et al., 2018). An extracellular *GDSL* lipase gene, *GDSL1* was identified in *Arabidopsis thaliana*. Loss of *AtGDSL1* amounted to increased susceptibility while overexpression of the gene was responsible for enhanced SA, JA, and ROS accumulation (Ding et al., 2020). An *NPR1*, *BnNPR1* gene was cloned from *B. napus*, which on overexpression resulted in increased resistance. This gene negatively regulated the JA pathway but had a positive modulation of SA (Wang et al., 2020).

Yield penalty is a serious issue caused by the ectopic expression of defense-related genes, so an *S. sclerotiorum*-induced promoter needs to be developed. In this line, Lin et al. (2022) developed *pBnGH17^D7^* which overcame this issue. It was also used for host-induced gene silencing control of the disease. Foliar application of *Verticillium dahliae Aspf2*-like protein induced many defense-related compounds in *B. rapa* and possessed a great ability to increase the SSR resistance (Jiang et al., 2022). In angiosperms, guaiacyl monolignol is converted to syringyl monolignol with the help of Ferulate-5-hydroxylase. Resistance and biomass recalcitrance are reportedly affected by the monolignol ratio. CRISPR/Cas9-mediated knocking of the *F5H* gene decreased the S/G ratio thereby enhancing the defense response (Cao et al., 2022).

## Downy mildew

Downy mildew (DM) is caused by a biotrophic oomycete, *Hyaloperonospora brassicae* (formerly *Hyaloperonospora parasitica*). Butler (1918) reported the disease, and it is found to be well spread in the major Brassica-growing regions of the world such as Canada, Australia, Europe, China and India (Saharan, 1992). All above-ground parts are affected by this seed-borne disease and the seedling stage is the most susceptible stage of all. Severe yield reduction has been reported from the adult plant stage on both floral and non-floral parts (Thines and Kummer, 2013; Meena et al., 2014b). Yellow or yellow-brown chlorotic lesions are visible on the upper surface of leaves, while the under surface has a prominent white powdery growth (Van De Wouw et al., 2016). Kaur et al. (2011b) reported that the severity of *A. candida* increased when co-infected with *H. brassicae* (**Figures 3G, H**). Multiple reports of the co-existence of *A. candida* and *H. brassicae* have been done in *B. juncea* in the Indian subcontinent, thus the development of resistant cultivars for both pathogens is essential to prevent yield losses (Saharan et al., 2016b; Mehta et al., 2018; Inturrisi et al., 2021).

*HaRxL106* is an effector secreted by *Hyaloperonospora arabidopsis* which interacts with the *Arabidopsis* RADICAL-INDUCED CELL DEATH1 (*RCD1*). It inhibits the transcription of both defense-related and salicylic acid-triggered genes. Along with that, Mut9-like kinases (*MLKs*) also play a significant role in modulating the SA levels as SA-induced defense genes were highly expressed in mlk1,3,4 triple mutants (Wirthmueller et al., 2018). Another effector from the *RxLR* super family; *HaRxLL470* interacted with *HY5* in the host which was responsible for photomorphogenesis regulation. It is a vital protein for the activation of defense-related genes and the effector binds with the DNA of *bZIP* transcription factor in *HY5* gene (Chen et al., 2021). Deb et al. (2018) characterised two effectors, *PsAvh73* and *HaRxL23* from *Phytophthora sojae* and *H. Arabidopsis*, respectively. These genes were found to be expressed very early during infection and suppressed PTI in Tobacco and ETI in Soybean. When the effector gene was constitutively expressed in *Arabidopsis*, it was found to impart resistance to these effectors. JA and SA signalling pathways are important for disease resistance in plants. An effector, *HaRxL10* attacks JA signalling by targeting the JAZ3 (transcriptional repressor) and finally attenuates the SA signalling which is detrimental for oomycetes. It functionally resembles the bacterial toxin coronatine that mimics the jasmonic acid signalling *via* TTSS. This further signifies the vulnerability of the plant defense system due to SA-JA crosstalk (Anderson et al., 2019).

Caillaud et al. (2012) observed the sub-cellular compartments of host mesophyll during haustorial growth. Huge changes occurred in the tonoplast which is located close to the extra-haustorial membrane. *HaRxL17* was in close association with tonoplast in un-infected cells and was found to be localised near the extra-haustorial membrane in case of infected ones. This establishes it as a potent effector which enhanced disease susceptibility. *HaRxL21* interacted with Topless protein (TPL) which is an *Arabidopsis* transcriptional corepressor. The interaction occurs at the C-terminus EAR motif and was proven to be essential for virulence as it mimics the recruitment of TPL to transcriptional repression sites (Harvey et al., 2020). The plant is well connected in a cell-to-cell manner *via* plasmodesmata, but it is not beneficial for it to keep them open in infected tissue as isolation of infected cells is a prerequisite for avoiding their transmission to healthy cells. Thus, the pathogen targets this isolation mechanism and keeps the plasmodesmata open in the host tissue. *HaRxL77* was reported to suppress the flg22-induced ROS response and promote hypermobility through manipulation of plasmodesmatal permeability. The study opens a new avenue that should be looked at, for plant-pathogen interaction (Liu et al., 2022). Dunker et al. (2020) reported an sRNA (*Hpas*RNA), which utilises the Argonaute (AGO)/RNA-induced silencing complex of the host for causing disease. The transgenic Arabidopsis inhibiting *Hpas*RNAs and atago*1* mutants were used to determine their combined role in virulence. Though the role of sRNA is evidently proven in virulence, the cross-kingdom RNAi (ck-RNAi) mechanism must be explored.

Fourteen isolates of *Peronospora parasitica* were tested on the *B. napus* cultivar Cresor and it was found resistant to all. This resistance was shown to be conferred by a single dominant gene. When two homothallic isolates which were avirulent on the cultivar were combined, it led to virulence (Lucas et al., 1988). Dickson and Petzoldt (1993) reported that seedling stage resistance in broccoli for downy mildew is independent of the mature-plant stage. As the resistance varied between different stages, the selection must be done at the mature stage rather than the former. An analysis was done to check the response of the downy mildew attack on *B. napus* and two accession lines were deemed to be resistant viz., RES-26 and RES-02. The resistance for isolate P003 was governed by two independent dominant genes in RES-02 and one partially dominant gene in RES-26. Another isolate R1 was inoculated on RES-02 and the resistance pattern was found to be incompletely dominant. The genes for resistance against P003 and R1 were either identical or closely linked (Nashaat et al., 1997). Coelho et al. (1997) identified a single dominant *R* gene (*Pp523*) that is responsible for imparting resistance to broccoli plants. This locus *Pp523* was situated on C8 and was the first gene to be identified to confer resistance to adult plants against *P. parasitica*. The flower colour character was found to be on C3, and the newly prepared map was denser than the earlier ones. This information can be used to perform map-based cloning of *Pp523* (Carlier et al., 2011). The syntenic region was located at the top arm of chromosome 1 in *A. thaliana* (Farinhó et al., 2007).

Jensen et al. (1999) evaluated 20 DH broccoli lines and found br8 and br9 lines to allow fewer conidia production by 50-70% as compared to susceptible ones. The br9 line was somewhat more uniform in resistance response than br8, in which a sort of isolate-specificity was found. 31.8% and 45.8% variations were observed in conidia production and sporulation score, respectively. Recurrent selection for partial resistance in the early generation is an effective method for selecting resistant lines against downy mildew. A resistant DH line (from Everest) was crossed to the susceptible (from Marathon). All F_1_ plants were found to be resistant and a 9:7 resistant to susceptible ratio was observed in the F_2_ generation. This confirmed the gene’s dominant nature, which can be incorporated in F_1_ hybrids and commercially released (Wang et al., 2001). An NBS-LRR gene, *RPP8* was identified and cloned by McDowell et al. (1998) and they did a comparative study of alleles in susceptible (*Col-0*) and resistant (*Ler-0*) accessions. In *Ler-0*, the *RPP8* haplotype had a functional gene along with non-functional *RPHA8*. On the other hand, the rpp8 locus had a single chimeric gene in *Col-0* accession. McDowell et al. (2005) mapped an adult resistance gene, *RPP31* on chromosome no. 5 of *A. thaliana*. A SA degrading transgene, NahG and mutation leading to loss of function in defense-related genes such as *PAD4*, *NPR1*, *PBS3* and *RAR1* were able to suppress the adult-plant resistance. Three white rust resistance-conferring loci (*RAC1*, *RAC2* and *RAC3*) were mapped and identified using two *Arabidopsis* accessions (ksk-1 and ksk-2). The two *P. parasitica* resistance genes *RPP8* and *RPP9* were found to be closely attached to *RAC3* and *RAC1*(Borhan et al., 2001). The *RPP5* gene in *A. thaliana* is responsible for downy mildew resistance and, it was positionally cloned by Parker et al. (1997). It encodes a protein with an NBS-LRR that resembles N and L6 proteins coded by *R* genes in plants. *RPP5* produces a single transcript against the N and L6 which uses alternative splicing to produce truncated proteins. The terminal segment of gene looks like the cytoplasmic domain of Drosophila Toll and mammalian interleukin1-transmembrane receptors (TIR). 52 germplasm accessions of *B. oleracea* and its family members were evaluated for DM resistance. A recessive gene was involved in resistance which goes against the earlier studies in favour of the dominant *R* gene. This may be because the isolate of *P. parasitica* was collected from the *B. napus* field (Carlsson et al., 2004).

Four tightly linked genes were identified on the RPP1 region of chromosome no. 3 in Wassilewskija accession of *A. thaliana*. Out of these four, three (*RPP1-WSSA*, *RPP1-WSSB*, *RPP1-WSSC*) were found to encode NBS-LRR. Previously, the resistance was thought to be conferred by the *RPP1* gene, but all three genes acted as complex loci and were responsible for resistance against multiple pathogen races (Botella et al., 1998). The *RPP-13* locus in *Arabidopsis* had a variation in its LRR domain which was instrumental in resistance against multiple races of *P. parasitica*, previously thought to be conferred by different *R* genes. *RPP13*-*Nd* was responsible for resistance against 5 isolates of the pathogen and *RPP13*-*Rld* recognised some other specific isolates (Bittner-Eddy et al., 2000). An orthologue of *RPP5*; *RPP4* was identified in *Col-0* accession and found to provide resistance against Emoy2, Emwa1 and Noco2 races of *P. parasitica*. It required the action of 12 defense compounds such as *Sa*, *EDS1*, *PAD4*, *DTH9*, *PBS2*, *PBS3*, *SID1* and *SID2*. Its expression at the cotyledonary stage was inhibited by mutations in *rps5-1*, *ndr1* and *npr1* but, no such effect was seen at adult plant stage (Van Der Biezen et al., 2002). *RPP2A*/*RPP2B* is a specific *R* gene which recognises *P. parasitica* isolate Cala2, mutational analysis and map-based cloning were done to study effector-receptor interaction. When this *R* gene was transferred into a new plant variety, it conferred complete resistance (Sinapidou et al., 2004). Yu et al. (2009) used MQM and interval mapping to construct an improved genetic map for *Brassica rapa* ssp. *pekinensis*. This pointed towards a major QTL on A08 and a major gene that imparted seedling resistance.

Yu et al. (2011) identified two microsatellite simple sequence repeat markers (*kbrb058m10-1* and *kbrb006c05-2*) and one sequence-characterised amplified region marker (*SCK14-825*) being closely linked to seedling resistance QTL (*BrDW*) located on chromosome A8 in *B. rapa* ssp. *pekinensis*. Extending their work, four major QTLs, *sBrDM8*, *yBrDM8*, *rBrDM8* and *hBrDM8* were mapped by Yu et al. (2016) for seedling, young plant, rosette and heading stages, respectively on chromosome A08. Two minor QTLs, *hBrDM6* on A06 and *rBrDM6* on A04 were also found to be active at the heading and rosette stage. Downy mildew resistance was imparted by a single locus, dominant gene *Ppa3* in cauliflower plants. 13 polymorphic markers were identified between two parental lines ‘Pusa Himjyoti’ and ‘BR-2’, out of which six were RAPD and seven were ISSR. Finally, a linkage map was constructed based on 120 F_2_ plants (Singh et al., 2012). Two inbred lines, RS1 and SS1 were selected from Chinese cabbage for downy mildew resistance and susceptibility, respectively. Evaluation for resistance was done in F_1_, F_2_ and BC_1_F_1_ populations and *BrRHP1* was identified as a dominant single locus. Two molecular markers, *BrPERK15A* and *BrPERK15B* were developed along with an RAPD marker that is closely attached to *BrRHP1* (Kim et al., 2011). Monteiro et al, 2005 checked the inheritance pattern for DM resistance at the cotyledonary and adult plant stage in *B. oleracea* var. *tronchuda*. For the cotyledonary stage, the F_2_ of the cross between resistant and susceptible varieties segregated in the ratio of 15:1, indicating that the trait is controlled by two duplicate dominant genes. The F_2_ adult plant stage segregated in the ratio 3:1, signifying a single dominant gene. *B. napus* was transformed by the *Agrobacterium* mediation and a bacterial catalase gene *katE* was introduced in the host chloroplast. The transformed and untransformed plants were checked for both downy mildew and powdery mildew resistance. The growth of fungi was seriously hampered in the transgenics and enzymes such as catalase, polyphenol oxidase and peroxidases were constitutively expressed, thus providing resistance to both diseases (El-Awady et al., 2008). Liu et al. (2021) evaluated forty members of Chinese cabbage CC-NBS-LRR. The phylogenetic relationship of CC-NBS-LRR was analysed in *A. thaliana*, *B. rapa* and *Oryza sativa*. They were also classified based on their conserved domains and role of BrCC-NBS-LRR was established in pathogenesis-related defense. Finally, expression profiling was done on both short-duration and long-duration basis.

## Conclusion

Effectoromics is an emerging concept which can be used to identify the pathogen and establish a phylogenetic relationship between members of the same species or across a larger group. It involves a high-throughput functional genomic way for examining plant genomes to find new *R* genes in cultivated brassica and its relatives. The cognate *R* gene interacting with the effector of the pathogen once identified can be introgressed in the high-yielding susceptible varieties. The *R* genes mostly belong to NBS-LRR class of protein-encoding genes and are responsible for narrow-spectrum resistance which is overcome by more virulent races. The introgression is always not without challenges, as sometimes the *R* genes are closely linked with other non-desirable agronomic traits or in some instances, *R* genes for biotrophs can behave as pathogen targets (S genes) for necrotrophs. The narrow spectrum nature of this resistance can be overcome by overexpressing the defense-related genes which form the basis of broad-spectrum resistance and relies on hormonal signalling pathways and strengthening of host tissues rather than targeting or interacting with the pathogenicity factors (effectors). There have been many studies conducted on modulating the expression of these genes that leads to resistance. Transgenics go beyond the conventional search of resistance sources in the host family. Many genes that are transferred from across the species, genera, and in some cases cross-kingdoms as well have been able to confer resistance. Thus, for developing a robust disease management strategy, identifying essential genes conferring the pathogenicity and the role of HSTs in each host-pathogen system for identifying the *R* gene sources, is a must. A better analysis of reaction of transgenics with its biotic and abiotic environment is also essential to check its compatibility with other control measures such as biocontrol. This review focuses on three major concepts: fungal effectors, *R* genes/QTLs and defense-related genes and transgenics so that in the future better resistance breeding modules can be developed.

## Author contributions

PR wrote the draft. PKR and LP edited the manuscript and provided critical review. All authors contributed to the article and approved the submitted version.
